# Ellipsoid Zone Integrity and Visual Acuity Changes during Diabetic Macular Edema Therapy: A Longitudinal Study

**DOI:** 10.1155/2021/8117650

**Published:** 2021-10-07

**Authors:** Lucy J. Kessler, Gerd U. Auffarth, Dmitrii Bagautdinov, Ramin Khoramnia

**Affiliations:** ^1^Department of Ophthalmology, University of Heidelberg, Heidelberg 69120, Germany; ^2^HEiKA–Heidelberg Karlsruhe Strategic Partnership, Heidelberg University and Karlsruhe Institute of Technology (KIT), Karlsruhe 76131, Germany

## Abstract

**Purpose:**

Ellipsoid zone (EZ) integrity is identified as a potential biomarker for therapy surveillance and outcome prediction of visual acuity (VA). However, only a few studies report long-term results of over 1 year of clinical and anatomical changes in patients with diabetic macular edema (DME). This study is aimed at describing the long-term VA and anatomical outcomes in spectral domain optical coherence tomography (OCT) (relative ellipsoid zone reflectivity ratio, central macular thickness, and volume) in patients with DME treated with antivascular endothelial growth factor (anti-VEGF) therapy. Furthermore, we studied the correlation between EZ integrity and changes in visual acuity.

**Methods:**

71 eyes of 71 patients were included in this retrospective study. Clinical characteristics were reviewed yearly. OCT data were assessed at baseline and after 1, 3, and 5 years. EZ parameters were quantified automatically. OCT parameters and visual outcome were correlated and analyzed in multivariable regression models.

**Results:**

EZ reflectivity ratio correlated with functional outcome in DME patients from baseline to fifth year at all time points (for all *p* < 0.05). EZ reflectivity improved the most in the first year of treatment (0.68 to 0.75; *p* < 0.05) and declined gradually until year 5 of therapy (0.71; compared to baseline *p* > 0.05). Similarly, best VA was achieved after 1 year (0.40 logarithm of the minimum angle of resolution (logMAR) to 0.28 logMAR; *p* < 0.001) and declined gradually until year 5. Final VA in year 5 was comparable to baseline (0.45 logMAR, compared to baseline *p* > 0.05). Together with baseline VA, baseline EZ parameters did predict VA outcome after 1 year (*p* < 0.05). Concordantly, VA and EZ parameters from year 1 were associated with VA outcome in year 2.

**Conclusion:**

This study described the long-term course of EZ changes during anti-VEGF treatment in DME patients. In addition, our results underlined the potential of EZ parameters as novel OCT biomarkers for prediction of VA outcomes during therapy.

## 1. Introduction

Center involved diabetic macular edema (DME) is a sight threatening manifestation in patients with diabetic retinopathy [1–3]. Intravitreal antivascular endothelial growth factor (anti-VEGF) injection has become the standard of care in preventing further vision loss [4, 5]. Response to anti-VEGF treatment is evaluated by clinical and morphological parameters in optical coherence tomography (OCT) [6, 7]. To date, it has been difficult to determine individual response, disease activity, and potential visual preservation over time. In the past, OCT parameters in clinical settings have been limited to “global” measurements of retinal thickness and macular volume. From recent research, it appears that novel OCT biomarkers can be used to better understand individual therapy response, disease progression, and improve treatment [8] One of these emerging OCT biomarkers is the relative ellipsoid zone reflectivity ratio (EZR) [9]. In healthy eyes, the external limiting membrane (ELM), ellipsoid zone (EZ), and retinal pigment epithelium (RPE) are represented in OCT as hyperreflective bands. EZ is defined as the hyperreflective band posterior to the ELM, and its hyperreflectivity is assumed due to high mitochondrial density in the inner segments of photoreceptor cells, indicating the vitality of these photoreceptors [9]. Changes of optical reflectivity of EZ have been observed in retinal pathologies [10–12]. Previous studies demonstrated a correlation of recovery of ellipsoid zone and visual acuity in retinal diseases [10, 13–17]. In diabetic macular edema, sequential restoration of EZ was observed after one year of anti-VEGF treatment [18]. However, the long-term outcome of EZR during anti-VEGF treatment remains unknown. Additionally, in several studies, the quantitative analysis of EZR was laborious and time consuming as the measurements were manually obtained and mostly at a limited number of regions of interest [16, 19, 20]. The aim of this study is to evaluate the ellipsoid zone outcome (EZR and EZ-RPE distance) during a 3- to 5-year follow-up of DME patients under anti-VEGF therapy and to find potential correlation with the visual acuity outcome beyond 1 year after treatment initiation. Our analysis of the EZ characteristics was greatly facilitated by an automated quantitative examination of 27 regions of interest of fovea-centered OCT B-scan at each time point. The evaluation is therefore objective and comprises 4833 measurements in total.

## 2. Materials and Methods

This was a retrospective study conducted at the University Eye Hospital of Heidelberg, Germany. Local ethics committee approval was obtained from the University of Heidelberg. All study protocols adhered to the tenets of the Declaration of Helsinki. This study was registered on the German Clinical Trial Register (registration number: DRKS00024399).

### 2.1. Study Cohort

We reviewed patients with treatment-naive DME who began anti-VEGF therapy between 2010 and 2018 with a minimum of 3-year follow-up period at our hospital. 5-year data was available from 37 out of 71 patients. VA was assessed yearly. OCT parameters were quantified at baseline and years 1, 3, and 5. These time points were chosen because several real-world studies with long-term follow up in patients with DME showed that most VA gain and structural changes were noticed at year 1, but VA worsening was observed after 3 years [21–23]. Therefore, evaluating OCT parameters changes at third year may provide more insight in the changes of structural OCT between the year 1 and year 5.

Exclusion criteria included age younger than 18 years, retinal or glaucoma surgery before the first anti-VEGF injection, amblyopia, uveitis, and uncontrolled glaucoma. Presence of other retinal diseases associated with macular edema such as retinal venous or arterial occlusive disease, severe epiretinal membrane, alterations of outer retinal layers, like drusen, pigment epithelium detachment, and EZ atrophy due to age-related macular degeneration also led to exclusion. Refractive error of more than 6 diopter spherical equivalents, lack of OCT-scans at any time point, or OCT imaging of low quality or signal strength (<30/35 with 35/35 being the best signal to noise ratio) that impaired analysis were excluded. Patients who received anti-VEGF injections in other clinics were excluded as well. If both eyes were eligible for study inclusion, as study eye, we chose the eye with the worse best corrected visual acuity at baseline.

Treatment initiation was our baseline point when patients began to receive monthly intravitreal anti-VEGF injections (ranibizumab, aflibercept, or offlabel bevacizumab). This was followed by retreatment on the basis of treat and extend regimen at the discretion of the treating ophthalmologist. The treatment decision was derived from the German or European guideline for treatment that was valid at that time [6, 24–26]. In the past, treat and extend regimen has been continuously modified due to new findings or in the attempt to further reduce injection burden. Some changes in injection scheme also applied to our study population from 2010 to 2018. For example, as early as 2010, injection intervals were extended or shortened in a fixed 2-week scheme. However, we subsequently also allowed a shortening or extension of interval of only 1 week in cases when overall treatment response was difficult to determine to allow a closer observation of disease activity. Therefore, we compared baseline statistics of the 3-year and 5-year cohorts to confirm nonsignificant differences despite of different follow-up times. 31 patients received additional dexamethasone implants during therapy. All patients underwent a comprehensive ophthalmologic examination at each visit, which included measurement of the best-corrected visual acuity (BCVA), slit-lamp biomicroscopy, indirect funduscopy, and spectral domain OCT (Spectralis, Heidelberg Engineering, Heidelberg, Germany). At each visit, we obtained the patient's latest HbA1c serum level.

### 2.2. Optical Coherence Tomography Acquisition and Analysis

#### 2.2.1. Image Acquisition

Images were obtained at each visit using the Spectralis Spectral Domain OCT with HeyEx software, versions 5.3.0.7 to 6.3.2.0 (Heidelberg Engineering GmbH, Heidelberg, Germany). We used a 6 mm × 6 mm macular cube line scan protocol to obtain the image data. The scan recorded at baseline was set as reference to ensure all subsequent OCT scans were acquired at precisely this location. The horizontal B-scan through the foveola was extracted for further analysis. All scans were applied in high-resolution mode (512 pixels along the *x*-axis) and an automated averaging of 9 frames for each line scan. Central macular thickness (CMT) and macular volume (MV) were obtained from device-integrated software.

#### 2.2.2. Image Processing and Analysis

**Figure 1:**
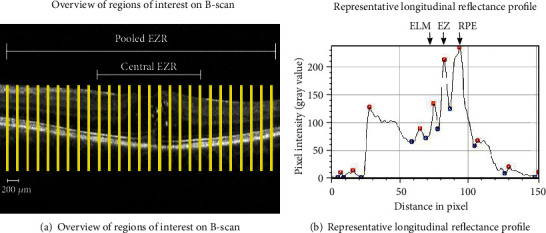
(a) Overview of regions of interest (ROI). In total, 27 measurements in 200 *μ*m distance of each OCT B-scan were obtained and analyzed. (b) shows a representative longitudinal reflectance profile at each ROI. Pixel intensity values ranged from 0 (black) to 255 (white) on gray scale. Maxima values (“peaks”) are represented by red dots and minima values between the peaks that are represented by blue dots.

Logarithmic-transformed display of OCT was exported as tagged image file format (TIFF) using the integrated Heidelberg Eye Explorer Software, version 1.10.4.0 (Heidelberg Engineering GmbH, Heidelberg, Germany). Prior to image analysis, image registration and signal normalization were applied using Fiji software, version 2.1.0/1.53c (US National Institutes of Health, Bethesda, US. https://imagej.net/software/fiji/) [27]. The foveola was used as center landmark for rigid image registration. OCT image with least speckle noise and best contrast was used as reference for histogram matching to normalize all OCT images. Longitudinal reflectance profile was obtained at every 200 *μ*m, thus resulting in 27 measurements of each OCT scan. The width of each region of interest (ROI) was set at 4 pixels (approximately 44 *μ*m). Reflectance profiles were taken in an automated fashion using a customized script including plot profile extraction in Fiji. This approach has been described elsewhere as a robust method to access EZ integrity [11, 19, 20]. Manual adjustment was not applied. Coordinates of peak values of the reflectance profile were stored as numeric values in a Microsoft Excel file (Microsoft Corp., Redmond WA, USA). Before retrieving and averaging peak values, plausibility of designated peak values was ensured by comparing plot profile and genuine OCT images. In reflectance profile, retinal pigment epithelium (RPE) is considered as the last hyperreflective band in the outer retina, whereas the ellipsoid zone (EZ) is the second hyperreflective band following the external limiting membrane (ELM). Relative ellipsoid zone reflectivity (EZR) was calculated as the ratio of EZ reflectivity to RPE reflectivity. The average value of EZR of the central 2000 *μ*m (in total 10 measurements) was considered as central EZR (c-EZR), and the average of all measurements (in total 27 measurements) was considered as pooled EZR (p-EZR) (Figure 1(a)). By averaging 27 measurements, shadowing effects caused by local pathologies such as hyperreflective dots or vessels that affect the optical reflectivity of underlying structures were mitigated. Only peak distance between EZ and RPE optical density more than 2 pixels (~22 *μ*m) was considered as two distinct peaks. Any peak distance below that was considered a partial EZ attenuation or atrophy was therefore not counted as a peak value and thus was not counted into the averaging calculation (Figure 1(b)). These thresholds were chosen according to the existing literature [28, 29].

### 2.3. Statistical Analysis

Snellen visual acuity was converted to logMAR for statistical analysis. For descriptive analysis, categorical data are presented as frequency and percentage (*n*; %); continuous data are shown as means with standard deviations (SD), median, and first and third quartile. Pearson's chi^2^ test and Mann–Whitney *U* test were applied to test for differences between independent groups. Spearman's Rho (*ρ*) was used for correlation analysis. For variance analysis, the Friedman test was applied to test for differences of continuous parameters across follow-up time points. Multiple variable linear regression analyses were performed to evaluate the effect of baseline parameters and mean changes between baseline and 1 year on visual outcome after 1 and 2 years, respectively. Multicollinearity, intercorrelation between independent variables that potentially can lead to model overfitting, was evaluated with variation inflation factor testing, and no models were run with variation inflation factor over 3 for any prediction. Statistical analysis was performed in IBM SPSS Statistics software version 27.0 (IBM Corp., Armonk, NY, USA); two-sided *p* < 0.05 was considered as statistically significant.

## 3. Results

### 3.1. Study Population

The mean age of the entire cohort was 59 (range: 42-79 years). In total, 45 patients were male (63.40%). 5-year data was available from 37 of 71 patients (71 eyes) (52%). At presentation, all eyes received the first intravitreal injection of one of three anti-VEGF medications: 74.60% got bevacizumab as first injection, 19.70% got ranibizumab, and 5.60% received aflibercept. During the observation period, between 2010 and 2018, the treatment guidelines were occasionally updated. Therefore, we compared the baseline characteristics of patients with and without 5-year data to confirm that both subgroups were comparable at baseline despite the difference in follow-up time (Table 1). There was no significant difference in the distribution of sex, age, and HbA1c serum level in both cohorts (*p* > 0.05). The number of patients who received laser treatment (focal or panretinal laser coagulation) prior to first injection and patients with pseudophakic study eyes was similar in both groups (*p* > 0.05). In the first year, the 3-year group received on average two more injections than the 5-year group (5-year group mean: 4.97; 3-year group: 7.00; *p* = 0.004). However, injection frequency per year was not significantly different in both groups. During observation time, the number of received dexamethasone implants was not significantly different in both groups (*p* > 0.05). Overall, baseline characteristics were similar in both groups.

### 3.2. Visual Acuity, Central Retinal Thickness (CRT), and Central Macular Volume

**Figure 2:**
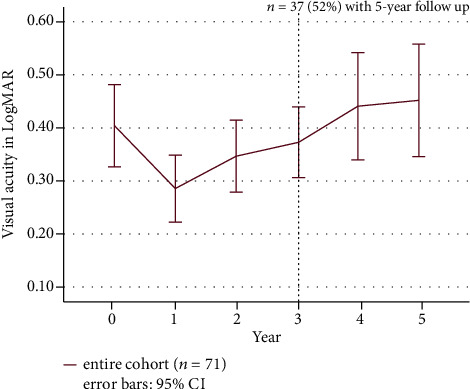
Mean visual acuity changes in logMAR from baseline to fifth year. Purple line represents the entire cohort (*n* = 71 until year 3). 4-year and 5-year data were available from 37 patients (dotted line). The 95% confidence interval is shown as error bars. Mean VA improved most in the first year. Improved mean VA was maintained until year 3. After 4 years, mean VA was worse than baseline VA despite continued therapy.

**Figure 3:**
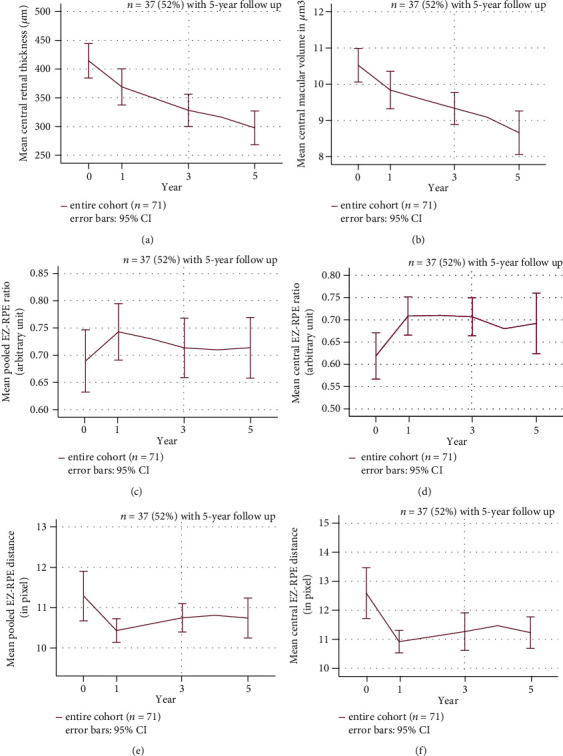
Longitudinal changes of OCT parameters from baseline to fifth year. Purple line represents the entire cohort (*n* = 71 until year 3). The 95% confidence interval is represented by error bars. (a) Central retinal thickness and (b) central macular volume decreased significantly during continuous therapy (*p* < 0.05). (c, d) show mean pooled and central EZ-RPE reflectivity ratio changes. (e, f) demonstrate mean pooled and central EZ-RPE distances.

VA was assessed yearly. OCT parameters including EZR and EZ-RPE distance, central macular thickness, and macular volume were retrieved at the start of treatment and after 1, 3, and 5 years. Changes of VA from treatment initiation to fifth year for the entire cohort are shown in Figure 2. Mean VA improved significantly from 0.40 logMAR (SD: 0.33) at baseline to 0.28 logMAR after 1 year (SD: 0.27; *p* < 0.001). The improvement was maintained until year 3. After 4 and 5 years, mean VA declined to 0.44 logMAR (SD: 0.30) and 0.45 logMAR (SD: 0.32), which was comparable to baseline VA (for 4 and 5 years: *p* > 0.05 compared to baseline).  Figure 3(a) represents the mean changes in CRT. Reduction of CRT was significant for all follow-up time points compared to baseline (for all time points: *p* < 0.05). Overall, CRT was reduced by approximately 46 *μ*m after 1 year compared to baseline (from 414 *μ*m (SD: 127.12) to 368 *μ*m (SD: 132.96), *p* < 0.05). CRT reduced continuously until year 5 to 297 *μ*m (SD: 88.15; *p* < 0.05). Mean central macular volume was 10.52 *μ*m^3^ at baseline and significantly decreased at 1- (9.84 *μ*m^3^; SD: 2.19; *p* < 0.001) and 3-year follow-up (9.33 *μ*m^3^; SD: 1.79; *p* < 0.001). Mean central macular volume at year 5 was 8.66 *μ*m^3^ and was significantly lower than at baseline (*p* < 0.001) (Figure 3(b)).

### 3.3. EZ-RPE Reflectivity Ratio and EZ-RPE Distance

Following initiation of anti-VEGF therapy, mean p-EZR improved from 0.68 (SD: 0.17) to 0.75 (SD: 0.15) in the first year (*p* < 0.001) and declined gradually to 0.71 (SD: 0.17) after 5 years (compared to baseline: *p* > 0.05) (Figure 3(c)). Mean c-EZR improved from 0.62 at baseline (SD: 0.22) to 0.71 (SD: 0.18) in year 1 for the entire cohort (*p* < 0.001). The improvement of c-EZR was maintained at year 3 (0.71, SD: 0.18) and declined in year 5 (0.69, SD: 0.20) after initiation of therapy. c-EZR at all time points was significantly higher than at baseline (for all time points *p* < 0.05) (Figure 3(d)). Baseline pooled EZ-RPE distance significantly reduced during therapy from 11.29 pixel (SD: 2.60) at baseline to 10.43 (SD: 1.24) at year 1 (*p* < 0.01) and 10.74 (SD: 1.49) at year 5 (*p* ≤ 0.01) (Figure 3(e)). Baseline central EZ-RPE distance reduced from 12.60 pixel (SD: 3.70) to 10.91 (SD: 1.63) after 1 year (*p* < 0.01), 11.00 (SD: 2.72) in year 3 (*p* < 0.05), and 10.91 (SD: 1.62) in year 5 (*p* < 0.05) (Figure 3(f)).

### 3.4. Correlation and Regression Analysis

At all time points, VA was significantly correlated to p-EZR (*ρ* ranged from -0.52 to -0.59, *p* < 0.05 at all time points). Correlation of c-EZR to VA was moderately higher than the p-EZR in the first and third year (*ρ* ranged from -0.52 to -0.65, *p* < 0.05 at all time points). Compared to EZR, pooled and central EZ-RPE distance showed lower yet significant correlation to VA at baseline and third year (Table 2). Mean VA improvement from baseline to year 3 was significantly correlated with improvement of c-EZR (*ρ* = −0.31, *p* < 0.01), central EZ-RPE distance (*ρ* = 0.26, *p* < 0.05), reduction of central retinal thickness (*ρ* = 0.40, *p* = 0.003), and improvement of macular volume (*ρ* = 0.40, *p* ≤ 0.001). Analysis with 5-year data revealed significant correlation between changes of VA, pooled, and central EZR after 5 years compared to baseline (*ρ* = −0.41 to -0.44, *p* < 0.05). c-EZR was best correlated to VA change. Correlation to VA change was not significant for mean change of EZ-RPE distance and mean macular volume changes (*p* > 0.05) (Table 3).

Regression models were controlled for age at treatment initiation, injection frequency in the first year, and baseline VA, which correlated strongly to VA improvements at all follow-up time points (*p* < 0.001). Mean change of EZ parameters was tested separately due to multicollinearity to predict change in VA at 1 year. Baseline pooled (*R*^2^ = 0.52, *p* < 0.001) and central EZR (*R*^2^ = 0.51, *p* < 0.001) as well as pooled (*R*^2^ = 0.52, *p* < 0.001) and central (*R*^2^ = 0.53, *p* < 0.001) EZ-RPE distance predicted change in VA after 12 months when controlled for the abovementioned variables. Likewise, for prediction of VA at year 2, when controlled for age at treatment initiation, injection frequency per year, and VA after 1 year, 1-year pooled (*R*^2^ = 0.52, *p* < 0.001) and central EZR (*R*^2^ = 0.53, *p* < 0.001) as well as 1-year pooled (*R*^2^ = 0.50, *p* < 0.001) and central (*R*^2^ = 0.50, *p* < 0.001) EZ-RPE distance predicted change in VA at year 2. We further evaluated the predictive value of age at treatment initiation for EZ restoration after 1 and 3 years. For this purpose, regression models were controlled for injection frequency in the first year, baseline VA, and baseline c-EZR or p-EZR. Adding age at treatment initiation as an additional independent variable, *R*^2^ increased from 0.40 to 0.46 (*p* < 0.001) for the prediction of c-EZR at 1 year and from 0.57 to 0.62 (*p* = 0.004) for the prediction of p-EZR after 1 year. It suggests that age at treatment initiation as a single variable explained approximately 5-6% of the variation of c-EZR and p-EZR after 1 year. However, this relationship was not significant for the prediction of 3-year EZ integrity with 1-year VA, injection frequency per year, c-EZR, and p-EZR (*p* > 0.05).

## 4. Discussion

We investigated the long-term changes of ellipsoid zone integrity during anti-VEGF therapy in DME patients in a real-world setting by evaluating the efficacy of EZ parameters in correlating and predicting VA outcomes at different time points. We followed patients for three years and five years after treatment initiation, in marked contrast to previous analyses which looked at functional and structural outcomes in diabetic patients over an observational period of mostly one year [7, 12, 30]. One earlier study did include clinical data of up to 4 years but did not include OCT parameters. [21] We focused on using OCT data. Previous studies included structural OCT parameters such as ellipsoid zone reflectivity, but mostly this was a qualitative analysis undertaken by masked graders, who categorized the grade of EZ disruption, or if a quantitative analysis was performed then it mostly was done manually [15, 18, 31]. Both qualitative and manual quantitative methods can be laborious and can potentially introduce bias from subjective judgment. Additionally, subtle differences in gray pixel values may be difficult to detect by masked graders. Recently, semi- or fully automated quantification methods were developed to facilitate objective analysis [32, 33]. In our study, we took advantage of image preprocessing and EZ parameters that we obtained in an automated way and included the average of 27 measurements of each fovea-centered OCT B-scan. Furthermore, we assessed both pooled and central EZ parameters to evaluate if the number and location of regions of interest affect the correlations between VA und EZ integrity. Notably, the correlation coefficient between central EZR and VA was slightly higher than pooled EZR at specific follow-up time points (years 1 and 3). The difference was considerably small. However, it hints that analyzing the central 2000 *μ*m with multiple measurements might be sufficient for EZ analysis. Involving the entire OCT B-scan for analysis did not lead to a better correlation between VA and EZ integrity. Changes of c-EZR from baseline to year 5 were more strongly correlated to change of VA from baseline to year 5 than CRT. This was already described by Shen et al. when they retrospectively evaluated EZ, ELM, CRT, and VA outcome in 40 DME patients [34]. Overall, correlation between EZ-RPE distance and VA was lower than correlation between EZR and VA. Changes of macular edema or retinal thickening which altered the retinal anatomy might have compromised distance measurements. It is questionable if volumetric quantification as performed by Ehlers et al. in en-face OCT projections can alleviate these limitations [10, 35]. Overall, we observed similar strength of correlations between EZ parameters and VA as in enface analysis used by Ehlers' group. In addition, Ehlers et al. used research-based software for EZ mapping that is not readily available to wide-spread clinical usage. In contrast, obtaining EZ reflectance profiles as performed in this study can be easily accomplished by clinicians by using the open-source platform Fiji.

Our results were in accordance with previous studies, which demonstrated that EZR was directly correlated to VA [14] and VA improvement in the first year of treatment [10, 16, 31, 36]. De et al. analyzed the integrity of the ELM and EZ in treatment-naive DME patients at baseline and after three injections [18]. Subsequent restoration of ellipsoid zone was observed after three injections. Otani et al. retrospectively studied cross-sectional OCT B-scans in 154 eyes with DME and demonstrated that the length of preserved ELM and photoreceptor inner segment/outer segment junction (currently termed EZ) at the fovea correlated with VA [37]. Ehlers et al. conducted a posthoc analysis of the VISTA study and analyzed the EZ integrity in 106 eyes of DME patients over approximately 2 years [10]. The authors concluded that in contrast to EZ parameters, subretinal fluid volume, central subfield retinal volume, and thickness were not significantly correlated to VA after 2 years. They found out that several EZ parameters such as central EZ-RPE volume and thickness were associated with VA throughout the follow-up period.

In our study, a significant correlation between EZ integrity and VA was observed until 5 years after initiation of therapy. We found that the most contributing predictor for VA outcome was baseline VA, a finding which has been reported previously by several authors [35, 38, 39]. Combined with EZ parameters, multivariable linear regression models showed that the visual outcome at 1 year can be predicted. Our study showed that age at treatment initiation has a predictive value for the EZ restoration at 1 year, which supports previous reports about increasing age being a negative predictive factor for final VA outcome [40]. These results underline the potential utility of EZ parameters as predictive OCT biomarkers to estimate VA changes after 1 year, which has been described in several retinal diseases associated with macular edema [10, 12, 18, 19, 35]. Further studies with prospective design and larger cohorts are needed to evaluate the predictive value of these biomarkers on overall outcomes and potential improvement in disease management in patients with diabetic macular edema.

The strengths of our study include the relatively long follow-up time of up to 5 years, the real-world setup, and the automated quantification of ellipsoid zone parameters. The limitations of our real-world study included its retrospective design, small cohort size, and nonstandardized VA assessment instead of the use of ETDRS charts. The study cohort is heterogeneous due to different onset of treatment initiation, follow-up time, and individualized combination of different anti-VEGF agents as well as dexamethasone implants. These are critical aspects of a real-world study that may negatively impact the main findings. The effects of different anti-VEGF agents on EZ integrity may differ, but this was not evaluated in the current study. Larger cohort size would enable stratification of patients into subgroups according to clinical or anatomical features which could prove to be helpful in characterizing the value of EZ parameters as biomarkers for individualizing the course of treatment. For instance, Chatziralli et al. observed that the extent of EZ restoration was dependent on the pattern of diabetic macular edema after 1 year of ranibizumab treatment in DME patients. Therefore, stratifying according to DME subtypes may enable better understanding of the retinal dynamics in treatment response. The lower injection frequency that we noted in the first year in the 5-year cohort was likely due to different treatment protocols at an earlier time. However, total injection frequency per year was nonsignificantly different in both 3-year and 5-year cohort, so that baseline characteristics are overall evenly distributed in the entire cohort. In addition, regression analysis was controlled for injection frequency.

In this study, we did not include other OCT biomarkers that might affect visual outcome in DME, such as hyperreflective foci or disorganization of the inner retinal layers (DRILs) [15, 41]. Nadri et al. found out that DRILs were correlated to the severity of diabetic retinopathy and EZ disruption [15]. Sun et al. confirmed that DRILs had predictive values for short-term VA outcome at 1 year [41]. However, including more biomarkers that are related to each other in a regression model might lead to an overfitted model, one that can be challenging to interpret and to evaluate the effect of single variables. Here, we focused on EZ changes and its direct correlation to VA changes; therefore, other OCT parameters were excluded.

All our patients received anti-VEGF agents as first line therapy; therefore, the isolated effect of dexamethasone on OCT biomarkers such as EZ integrity was not evaluated. EZ integrity has been identified as a positive predictor for VA gain in patients with treatment naive or refractory DME who received dexamethasone implants [42, 43]. Our findings support a positive correlation between EZ restoration and VA improvement. However, Zur et al. did not follow morphologic parameters after dexamethasone implantation; hence, it remains unclear how dexamethasone affects EZ restoration in eyes with treatment naive DME [42]. It is suggested that dexamethasone and anti-VEGF agents target different pathophysiological pathways [44]. The anti-inflammatory effect of dexamethasone may provide a better resolution of OCT biomarkers that presumably represent signs of retinal inflammatory response such as hyperreflective foci or serous detachment of neuroepithelium [45]. In our study, we observed a decline of EZ restoration after 3 years but did not further investigate the relationship between EZ integrity and other biomarkers. The potential association between EZ restoration and severity of retinal inflammatory signs in DME is an interesting aspect that has not been fully elucidated. Overall, further studies are needed to evaluate the long-term changes of VA and OCT biomarkers in DME treated with anti-VEGF or dexamethasone as first-line agents.

## 5. Conclusions

In summary, relative ellipsoid zone reflectivity ratio represents an additional potential biomarker to evaluate the course of anti-VEGF treatment in DME patients. Our results confirmed the relationship between EZ integrity and VA changes from baseline to year 5 and thus demonstrated the relationship beyond 1 year after initiation of therapy. However, further investigation is needed to evaluate the predictive value of EZ integrity as a biomarker on the overall outcome.

## Figures and Tables

**Table 1:** Demographics and characteristics for patients with 5-year data (*n* = 37) compared to patients with only 3 years of follow-up (*n* = 34).

Baseline variables	With 5-year data (*n* = 37)		Only 3-year data (*n* = 34)		*p*
	Mean (SD)	Median (Q1; Q3)	Mean (SD)	Median (Q1; Q3)	
Age (years)	57.54 (8.04)	57.00 (51.00; 63.00)	61.67 (8.80)	63.00 (54.00; 68.00)	0.051°
HbA1c (%)	7.19 (1.27)	6.95 (6.49; 7.88)	7.40 (0.96)	7.37 (6.76; 7.60)	0.886°
VA in logMAR at baseline	0.39 (0.29)	0.30 (0.15; 0.59)	0.42 (0.37)	0.37 (0.10; 0.58)	0.799°
Pooled EZ-RPE reflectivity ratio (arbitrary unit)	0.69 (0.17)	0.70 (0.59; 0.81)	0.67 (0.16)	0.68 (0.59; 0.81)	0.756°
Pooled EZ-RPE distance (in pixel)	11.24 (1.53)	11.04 (9.89; 12.27)	11.33 (3.43)	10,81 (10.07; 11.63)	0.475°
Central retina thickness (in *μ*m)	409.86 (133.22)	367.00 (312.00; 470.00)	419.30 (121.96)	370.50 (321.75; 489.75)	0.600°
Central macular volume (in *μ*m^3^)	10.55 (2.19)	9.93 (9.18; 11.53)	10.50 (1.70)	10.39 (9.10; 11.20)	0.756°
	*n* (%)		*n* (%)		
Male sex	26 (70.30)		19 (55.90)		0.229^†^
Laser treatment before treatment	15 (40.54)		9 (26.47)		0.315^†^
Pseudophakia before treatment	7 (18.91)		12 (35.29)		0.180^†^
Variables during observation					
Received dexamethasone implants	1.51 (2.90)	0.00 (0.00; 2.00)	1.62 (2.13)	0.50 (0.00; 3.00)	0.444°
Injections per year	5.11 (1.67)	5.08 (3.83; 6.20)	5.83 (1.75)	5.70 (4.27; 6.94)	0.777°
Injections in first year	4.97 (2.35)	5 (3.00; 6.00)	7.00 (2.88)	6.00 (5.00; 10.00)	*0.004*°

logMAR: logarithm of the minimum angle of resolution; p-EZR: pooled relative ellipsoid zone reflectivity; c-EZR: central relative ellipsoid zone reflectivity; EZ: ellipsoid zone; RPE: retinal pigment epithelium; SD: standard deviation; Q1: first quartile; Q3: third quartile. *p* values from °Mann–Whitney *U* test and ^†^Pearson's chi^2^ test. Values in italic front style denote statistical significance at the *p* < 0.05 level.

**Table 2:** Correlation analysis of visual acuity and OCT parameters at baseline, 1, 3, and 5 years. Spearman's correlation coefficient *ρ* between visual acuity and OCT parameter at each time point is shown. ^∗^*p* < 0.05; ^∗∗^*p* < 0.01; ^∗∗∗^*p* < 0.001.

		Visual acuity			
		Baseline	Year 1	Year 3	Year 5
Pooled EZR	Baseline	-0.52^∗∗∗^			
	Year 1		-0.56^∗∗∗^		
	Year 3			-0.52^∗∗∗^	
	Year 5				-0.59^∗∗∗^
Central EZR	Baseline	-0.52^∗∗∗^			
	Year 1		-0.65^∗∗∗^		
	Year 3			-0.56^∗∗∗^	
	Year 5				-0.55^∗∗∗^
Pooled EZ-RPE distance	Baseline	0.25^∗^			
	Year 1		0.26^∗∗∗^		
	Year 3			0.31^∗^	
	Year 5				0.24
Central EZ-RPE distance	Baseline	0.28^∗^			
	Year 1		0.23		
	Year 3			0.26^∗^	
	Year 5				0.27

**Table 3:** Correlation analysis of changes in visual acuity and OCT parameters between baseline to third year and baseline to fifth year. Spearman's correlation coefficient *ρ* between change of visual acuity and OCT parameter at each time point is shown. ^∗^*p* < 0.05; ^∗∗^*p* < 0.01; ^∗∗∗^*p* < 0.001.

Change of OCT parameters at the same time period	Change in visual acuity	
	Baseline to year 3	Baseline to year 5
Pooled EZR	-0.21	-0.41^∗^
Pooled EZR-RPE distance	0.21	0.33^∗^
Central EZR	-0.31^∗∗^	-0.44^∗∗^
Central EZ-RPE distance	0.26^∗^	0.21
CRT	0.40^∗∗^	0.34^∗^
Macular volume	0.40^∗∗^	0.07

## Data Availability

The data used to support the findings of this study are included within the article.

## References

[1] Ciulla T. A., Amador A. G., Zinman B. (2003). Diabetic retinopathy and diabetic macular edema: pathophysiology, screening, and novel therapies. *Diabetes Care*.

[2] Wong T. Y., Klein R., Islam F. M. A. (2006). Diabetic retinopathy in a multi-ethnic cohort in the United States. *American Journal of Ophthalmology*.

[3] Xie X. W., Xu L., Wang Y. X., Jonas J. B. (2008). Prevalence and associated factors of diabetic retinopathy. The Beijing Eye Study 2006. *Graefe's Archive for Clinical and Experimental Ophthalmology*.

[4] Mitchell P., Wong T. Y. (2014). Management Paradigms for Diabetic Macular Edema. *American Journal of Ophthalmology*.

[5] Stewart M. W. (2014). Anti-VEGF therapy for diabetic macular edema. *Current Diabetes Reports*.

[6] Schmidt-Erfurth U., Garcia-Arumi J., Bandello F. (2017). Guidelines for the management of diabetic macular edema by the European Society of Retina Specialists (EURETINA). *Ophthalmologica*.

[7] Chatziralli I., Kazantzis D., Theodossiadis G., Theodossiadis P., Sergentanis T. (2021). Retinal layers changes in patients with diabetic macular edema treated with intravitreal anti-VEGF agents: long-term outcomes of a spectral-domain OCT study. *Ophthalmic Research*.

[8] Markan A., Agarwal A., Arora A., Bazgain K., Rana V., Gupta V. (2020). Novel imaging biomarkers in diabetic retinopathy and diabetic macular edema. *Therapeutic Advances in Ophthalmology*.

[9] Tao L. W., Wu Z., Guymer R. H., Luu C. D. (2016). Ellipsoid zone on optical coherence tomography: a review. *Clinical & Experimental Ophthalmology*.

[10] Ehlers J. P., Uchida A., Hu M. (2019). Higher-order assessment of OCT in diabetic macular edema from the VISTA study: ellipsoid zone dynamics and the retinal fluid index. *Ophthalmology Retina*.

[11] Gong Y., Chen L. J., Pang C. P., Chen H. (2021). Ellipsoid zone optical intensity reduction as an early biomarker for retinitis pigmentosa. *Acta Ophthalmologica*.

[12] Tsai M. J., Cheng C. K. (2021). Patterns of ellipsoid zone change associated with visual outcome for diabetic macular oedema. *Clinical & Experimental Optometry*.

[13] Ciulla T. A., Kapik B., Grewal D. S., Ip M. S. (2021). Visual Acuity in Retinal Vein Occlusion, Diabetic, and Uveitic Macular Edema: Central Subfield Thickness and Ellipsoid Zone Analysis. *Ophthalmology Retina*.

[14] Maheshwary A. S., Oster S. F., Yuson R. M. S., Cheng L., Mojana F., Freeman W. R. (2010). The Association Between Percent Disruption of the Photoreceptor Inner Segment- Outer Segment Junction and Visual Acuity in Diabetic Macular Edema. *American Journal of Ophthalmology*.

[15] Nadri G., Saxena S., Stefanickova J. (2019). Disorganization of retinal inner layers correlates with ellipsoid zone disruption and retinal nerve fiber layer thinning in diabetic retinopathy. *Journal of Diabetes and its Complications*.

[16] Sharef N., Kassem R., Hecht I. (2021). Interdigitation and ellipsoid zones disruption correlate with visual outcomes among treatment-naive patients with diabetic macular edema. *Ophthalmic Research*.

[17] Ciulla T. A., Pollack J. S., Williams D. F. (2021). Visual acuity outcomes and anti-VEGF therapy intensity in diabetic macular oedema: a real-world analysis of 28 658 patient eyes. *The British Journal of Ophthalmology*.

[18] De S., Saxena S., Kaur A. (2021). Sequential restoration of external limiting membrane and ellipsoid zone after intravitreal anti-VEGF therapy in diabetic macular oedema. *Eye (London, England)*.

[19] Gin T. J., Wu Z., Chew S. K. H., Guymer R. H., Luu C. D. (2017). Quantitative analysis of the ellipsoid zone intensity in phenotypic variations of intermediate age-related macular degeneration. *Investigative Ophthalmology & Visual Science*.

[20] Toprak I., Yaylali V., Yildirim C. (2014). Decreased photoreceptor inner segment/outer segment junction reflectivity in patients with idiopathic epimacular membrane. *Eye (London, England)*.

[21] Granstam E., Rosenblad A., Raghib A. M. (2020). Long-term follow-up of antivascular endothelial growth factor treatment for diabetic macular oedema: a four-year real-world study. *Acta Ophthalmologica*.

[22] Van Aken E., Favreau M., Ramboer E. (2020). Real-world outcomes in patients with diabetic macular edema treated long term with ranibizumab (VISION study). *Clinical Ophthalmology*.

[23] Massin P., Creuzot-Garcher C., Kodjikian L. (2021). Real-world outcomes after 36-month treatment with ranibizumab 0.5 mg in patients with visual impairment due to diabetic macular edema (BOREAL-DME). *Ophthalmic Research*.

[24] BÄK B. (2015). *Arbeitsgemeinschaft der Wissenschaftlichen Medizinischen Fachgesellschaften (AWMF)*.

[25] Federfuhrendes R. (2013). Statement of the German Ophthalmological Society, the Retina Society and the Professional Association of German Ophthalmologists: treatment of diabetic maculopathy (April 2013). *Klinische Monatsblätter für Augenheilkunde*.

[26] Deutsche Ophthalmologische G., Retinologische Gesellschaft E., Berufsverband der Augenarzte Deutschlands E. (2020). Statement of the German Ophthalmological Society, the Retinological Society and the Professional Association of Ophthalmologists in Germany on treatment of diabetic macular edema: Situation August 2019. *Der Ophthalmologe*.

[27] Schindelin J., Arganda-Carreras I., Frise E. (2012). Fiji: an open-source platform for biological-image analysis. *Nature Methods*.

[28] Itoh Y., Petkovsek D., Kaiser P. K., Singh R. P., Ehlers J. P. (2016). Optical coherence tomography features in diabetic macular edema and the impact on anti-VEGF response. *Ophthalmic Surgery, Lasers & Imaging Retina*.

[29] Ugwuegbu O., Uchida A., Singh R. P. (2019). Quantitative assessment of outer retinal layers and ellipsoid zone mapping in hydroxychloroquine retinopathy. *The British Journal of Ophthalmology*.

[30] Granström T., Forsman H., Olinder A. L. (2016). Patient-reported outcomes and visual acuity after 12 months of anti-VEGF- treatment for sight-threatening diabetic macular edema in a real world setting. *Diabetes Research and Clinical Practice*.

[31] Serizawa S., Ohkoshi K., Minowa Y., Soejima K. (2016). Interdigitation zone band restoration after treatment of diabetic macular edema. *Current Eye Research*.

[32] Thiele S., Isselmann B., Pfau M. (2020). Validation of an automated quantification of relative ellipsoid zone reflectivity on spectral domain-optical coherence tomography images. *Translational Vision Science & Technology*.

[33] Etheridge T., Dobson E. T. A., Wiedenmann M. (2020). A semi-automated machine-learning based workflow for ellipsoid zone analysis in eyes with macular edema: SCORE2 pilot study. *PLoS One*.

[34] Shen Y., Liu K., Xu X. (2016). Correlation between visual function and photoreceptor integrity in diabetic macular edema: spectral-domain optical coherence tomography. *Current Eye Research*.

[35] Abraham J. R., Boss J., Babiuch A. S. (2021). Longitudinal assessment of ellipsoid zone mapping parameters in retinal venous occlusive disease with associated macular edema. *Journal of VitreoRetinal Diseases*.

[36] Chatziralli I., Theodossiadis G., Dimitriou E., Kazantzis D., Theodossiadis P. (2020). Association between the patterns of diabetic macular edema and photoreceptors' response after intravitreal ranibizumab treatment: a spectral-domain optical coherence tomography study. *International Ophthalmology*.

[37] Otani T., Yamaguchi Y., Kishi S. (2010). Correlation between visual acuity and foveal microstructural changes in diabetic macular edema. *Retina*.

[38] Choovuthayakorn J., Tantraworasin A., Phinyo P. (2021). Factors associated with 1-year visual response following intravitreal bevacizumab treatment for diabetic macular edema: a retrospective single center study. *Int J Retina Vitreous*.

[39] Choovuthayakorn J., Phinyo P., Tantraworasin A. (2021). Intravitreal anti-vascular endothelial growth factor therapy for diabetic macular edema in clinical practice of single center: three-year outcomes. *Ophthalmic Research*.

[40] Chatziralli I., Theodossiadis P., Parikakis E. (2017). Dexamethasone intravitreal implant in diabetic macular edema: real-life data from a prospective study and predictive factors for visual outcome. *Diabetes Therapy*.

[41] Sun J. K., Radwan S. H., Soliman A. Z. (2015). Neural retinal disorganization as a robust marker of visual acuity in current and resolved diabetic macular edema. *Diabetes*.

[42] Zur D., Iglicki M., Busch C. (2018). OCT biomarkers as functional outcome predictors in diabetic macular edema treated with dexamethasone implant. *Ophthalmology*.

[43] Meduri A., Oliverio G. W., Trombetta L., Giordano M., Inferrera L., Trombetta C. J. (2021). Optical Coherence Tomography Predictors of Favorable Functional Response in Naïve Diabetic Macular Edema Eyes Treated with Dexamethasone Implants as a First-Line Agent. *Journal of Ophthalmology*.

[44] Romero-Aroca P. (2010). Targeting the pathophysiology of diabetic macular edema. *Diabetes Care*.

[45] Ceravolo I., Oliverio G. W., Alibrandi A. (2020). The application of structural retinal biomarkers to evaluate the effect of intravitreal ranibizumab and dexamethasone intravitreal implant on treatment of diabetic macular edema. *Diagnostics (Basel)*.

